# Castration of Sexual Perverts*

**Published:** 1893-12

**Authors:** F. E. Daniel

**Affiliations:** Ed. Tex. Med. Journal, Austin, Tex.


### TEXAS MEDICAL JOURNAL.

ESTABLISHED JULY, 1885.

PUBLISHED MONTHLY.—SUBSCRIPTION $2.00 A YEAR.

VOL. IX AUSTIN, DECEMBER, 1893. NO. 6.

### Original Contributions.

### CASTRATION OF SEXUAL PERVERTS.*

BY F. E. DANIEL, M. D., ED. TEX. MED. JOURNAL, AUSTIN, TEX.

WITH a better knowledge of the laws of health, and a deeper research into the causes of diseases that most afflict mankind, great advance has been made in preventive medicine; and with each conquest the prospect broadens, reveals possibilities not before thought of, and to-day sanitation is generally recognized as a distinct science.

This progress unfortunately, however, has been made along the lines of physical disease mostly, for, notwithstanding the distinct recognition accorded to heredity as a factor in (if it is not the most prolific source of) disease, particularly mental disease, we know of little or nothing being done to lessen the evil of transmission of vice, disease, and the propensity to crime.

No fact is better established than that drunkenness, insanity, and criminal traits of character, as well as syphilis, consumption, and scrofula, may descend from parent to child. With the ex-

[* Under the title, "Should Insane Criminals or Sexual Perverts be Permitted to Procreate?" this paper was read at the Joint Session of the World's Columbian Auxiliary Congress—Section of Medical Jurisprudence—and the International Medico-Legal Congress, August 16th, 1893, and also before the American Medico-Legal Society, New York, October 11th, 1893, and published in the "Medico-Legal Journal" for December, and in the "Psychological Bulletiu," New York.—Advanced sheets kindly furnished by Hon. Clark Bell, Editor M.-L. Journal, President of the Congress.—ED.]

ception of improved hygiene in lunatic asylums, and more enlightened and rational treatment, nothing, or nearly nothing, is being done in the way of rational prophylaxis against a long list of maladies that destroy both body and mind. In no State are such restrictions put upon the privilege of marrying as are calculated to arrest the propagation of consumption, syphilis, insanity, drunkenness, and criminal propensity; nor is any other method resorted to, calculated to counteract, or lessen the degrading effects of hereditary transmission of these vices.

In Texas there are, in round numbers, four thousand insane people. The asylums, with an aggregate capacity of fifteen hundred, are always full. The law stipulates that cases of recent development (and, therefore, supposed to be amenable to treatment), shall have precedence over chronic, and, presumably, incurable cases. Although rich in resources, the State has not yet made provision for all of this unfortunate class, (and this is true of nearly all other American states). Should the order of admission be reversed or interfered with, the acute or recent cases, and still many chronic cases, would soon be where the latter are now, at home or in the jails. In Texas some are in the penitentiary, as will be seen presently, suffering punishment for crime. The wealth of all the Czars would not be adequate to provide asylum and medical treatment for the progeny of these people in fifty years from now; for, while insane people do not marry, those do in whom the disease exists undeveloped, and with the lower classes, particularly negroes, it is known that illicit intercourse is extremely common.

All medical men recognize the powerful influence of the sexual sense on human character and action. In the healthy person it dominates life, and is the great incentive to action, to the acquisition of property, the struggle for social eminence, and the foundation of a home. But how few of even the best informed physicians have more than a superficial knowledge of its aberrations, and the numerous anomalies that find expression in unnatural acts. How infinitely less is their knowledge of the causes that lead to such anomalies or perversion of that sense.

In comparative ignorance, then, both of the pathology, psychology, and etiology of sexual perversion, how little able are we to estimate the influence it exerts upon the mental and moral health, and the conduct of the unfortunates. We know that heredity is responsible for a large part of it; but whether perversion of the sexual sense, as expressed in unnatural methods of gratifying the desire be the *cause* or the *result* of disordered mind, we do not definitely know. Writers differ widely on this point, and it is, after all, a matter of individual opinion in a given case. Dr. Theodore Kellogg (*Reference Hand-Book of Medical Sciences*) says:

"The medical error of all ages has been to mistake symptoms for causes, and in no instance has this been more strikingly exemplified than in the misapprehension of the sexual manifestations of insanity. There is a widely prevalent belief, medical as well as popular, that a large proportion of all cases of mental derangement proceeds from natural or unnatural sexual indulgence. The symptom has here been mistaken for the cause of the disease. The scientific fact is, that perversion of the organic nature and appetites is a part of the very essence of insanity, and that of the sexual instinct, as the most fundamental, is the one most commonly involved. In most cases of insanity, increase, diminution, or perversion of the sexual appetite, exists at some stage of the disease, and frequently as one of the premonitory symptoms. Thus, the discovery of masturbation in the pubescent state is at once regarded as evidence of previous vicious indulgence, and the origin of the insanity is set down as found."

Notwithstanding this high authority to the contrary, no one at all acquainted with the subject will deny that masturbation (a perverted sexual sense) may, and frequently does, become a cause of mental alienation; and I can not subscribe to the belief that it is always a symptom of mental disease already existing. There is no doubt that habitual masturbation is often a manifestation of mental disease; nor, on the other hand, that it will lead to, and become a cause of, insanity. At puberty, when, often, the sexual instinct is more fully developed than the moral nature, many young persons practice masturbation, in whom, nor in whose family, no predisposition or hereditary taint exists; and when they become older, and can understand that it is disgraceful, and realize the tendency to moral and physical degeneration, they abandon it, and no evil consequences result. I may say I believe it is the experience and observation of nearly every physician that a majority of boys masturbate at some time during the development of the sexual system.

Then, in the state of our knowledge, who shall say when it is a *cause* and when an *effect*?

The subject of perversion of the sexual sense has, admittedly, not been studied in all its details. There is an innate repugnance to going deeply into investigation in a field where, from the glimpses given us by Krafft-Ebing, Charcot, Casper, Lydston, Kiernan, Parvin, and other investigators, we are sure to find so much revealed that is shocking to every sense of decency, disgusting and revolting. And I know of no attempt anywhere being made to either cure sexual perversion in males (except as to general treament for insanity in asylums), or to suppress its expression in unnatural gratification; our laws are defective, and do not deal intelligently with the subject. Certainly I know of nothing being done to arrest its descent to coming generations.

"It is a question," says Dr. Chaddock in the preface of that very remarkable book, *Psychopathia Sexualis* (quoting a German writer), "if it is justifiable to discuss the anomalies of the sexual instinct apart, instead of treating them in their proper place in psychiatry. As a rule, they are certainly only symptoms of a constitutional malady, or of a weakened state of the brain which manifest themselves in the various forms of sexual perversion."

I doubt if the propriety of a deeper, a most thorough study of this condition, so extensive throughout the world--this important factor in, and concomitant or manifestation of mental disease—the cause of so much human misery and degradation, can be longer questioned. It seems to me that the time has come, if progress in this field of medical study is to keep pace with the other branches, when a knowledge, not only of the various forms of sexual perversion, but of their cause, mode of development, progress and results, is *imperatively demanded.* It is a reproach to the medical profession that, as Dr. Chaddock says (*loc cit.*):

"Sexual anomalies, treated as they are in a distant manner in text-books on psychiatry are, for the physician, in greater part, *a terra incognita.* Exact knowledge of the causes and conditions of development of sexual perversion, and of their influence on hereditary constitutions, education, the impressions of every-day life and modern refined civilization, is the *prerequisite* for a rational prophylaxis of sexual aberration. * * * Without a careful study of the circumstances which attend the *development* of sexual anomalies, we should never be in a position to use effective therapeusis."

We may add, nor will we ever be in a position to do exact justice in case of trial before the courts for any sexual offense against the laws, either to the public, or to the accused, or to posterity.

The physician is often called upon to decide a case which hinges upon the mental state of an accused. Liberty, happiness, honor—life itself, to say nothing of posterity—depend, often, upon a medical examination; how humiliating, how worse than criminal to acknowledge that a perverted sense and its influence upon human actions are an unknown quantity to the medical profession. We often see it stated in criminal proceedings that "a medical examination was made and the man pronounced sound in mind," as if medical examinations were infallible, and that, too, in a field not only not explored, but confessedly as yet, nearly virgin soil. Krafft-Ebing's recent work (to which reference has already been made) is a startling revelation to many really well-read physicians, and his example should be followed till every sexual crime is understood, classified, and properly designated, and a penalty fixed by law to suit the crime.

Rape is defined to be sexual intercourse with a woman by force or against her will, or under the influence of a drug, or with a girl under (in some States 12, in others 14) years of age. The sexual act, or an attempt at it, by an adult with a small child, is more than rape; it is, alas, too often, murder in its cruelest form. (Some cases of so-called rape, it has been thought by the people of Texas, have not been adequately provided for in the penal statutes, notwithstanding rape is a capital offense.)

It is a maxim of law that an idiot, or an imbecile, or other irresponsible person, cannot commit a crime. Humanity revolts at the idea of punishing an irresponsible person, and in our courts every effort is made that our imperfect knowledge of mental disease will enable us to make, to discriminate in cases of evil-doing, between the responsible and the irresponsible. The ablest physicians, those having reputation in mental disease, are summoned as experts; but they often differ, there being no infallible rule or standard by which to measure responsibility—no certain test, for that of "knowing right from wrong" is about as definite as "the size of a piece of chalk." Right and wrong are arbitrary terms; matters of conscience and conscience, of education. What is wrong with one person and from one standpoint, may be right with another. Hence, injustice is, no doubt, necessarily often done, and discredit is brought upon the very name of medicine. Benjamin Vaughn Abbott (*Reference Hand-Book Medical Sciences*, page 122) says:

"The rude division into 'idiots' and 'lunatics' of two centuries ago, survives in jurisprudence to-day. * * * Jurisprudence has not possessed any peculiar means of studying the subject (insanity), but has been accustomed to follow the course of medical science, and to accept, sometimes, indeed, after long hesitation and inquiry, the results which skillful and experienced alienists have united in declaring established." * * *

(As has been shown, with regard to the phase of insanity under consideration, "following the course of medical science" is a case of "blind leading the blind," is it not?) The medical profession are still very far from thoroughly understanding the relation between, and the mutual influence of the sexual sense and the mind, the one upon the other; profoundly ignorant of that state that operates more largely, perhaps, than any other to produce irresponsibility (or, which state may be but the expression in acts of unsound mind), and beyond doubt, because of that ignorance many a criminal escapes the just penalty for his crime on the opinion of a medical expert that he is "insane"; while on the other hand, many really invalid, mentally (and therefore irresponsible), are held as criminals, and punished for acts which they commit in obedience to an impulse for which they are not responsible, and cannot control. Of course, in extreme cases a differentiation can be, and frequently is made; but there are so many grades and shades of mental unsoundness, produced by such a diversity of causes, and finding expression in such a variety of acts, that in our present knowledge it is impossible to draw the line; it is like differentiating a pig, a shoat and a hog.

Certain acts, are, however, *prima facie* evidence of insanity: thus, where a mother in cold blood kills her children. I take it that all acts contrary to reason and nature are expressions of an unsound mind. Who can doubt the insanity, even without a medical examination, of that poor shoe-maker's apprentice (*Psychopathia Sexualis*, p. 406,) who raped a goose, and on being caught in the act and arrested, innocently wanted to know if there was "anything the matter with the goose?"

At any rate, the dictates of humanity would give the perpetrators of such acts the benefit of the doubt, and treat them accordingly.

True, with regard to sexual crimes, a healthy person dominated by a powerful sexual impulse, may commit some act to shock a civilized community,—a rape, for instance. But even here, it is to be questioned if the inability to control the impulse, in the face of such powerful restraining influences as a certain knowledge of the fearful consequences which will follow such act, is not, in itself, evidence of irresponsibility? an indication of insanity? But were a sane man to commit rape it would likely be in accordance with, and not in violation of nature; he would select for his object an adult human being of the opposite sex, where intercourse would not be a physical impossibility. Were it otherwise he would not be a "sane man." In a case where a powerful man, especially a negro, who, in the South, at least, should have little excuse for unsatisfied sexual desire, amongst a race whose ideas of morality are crude, and virtue is not a striking characteristic, attempts to effect sexual intercourse with a small child of a different race,—a physical impossibility,—and that, too, in knowledge that if caught he will surely meet with a speedy and horrible death, it would be, in my opinion, *prima facie* evidence of an unsound mind, insanity in some degree. Still there may occur such cases, attended with circumstances, which, if they do not justify, at least afford excuse for punishment. It may be, as was the case in a notorious instance in Texas recently, that although the act (forcing entrance into the body of a white baby by a stout negro, by tearing the child limb from limb) was *prima facie* evidence of perverted sexual instinct, and *ergo*, of unsound mind, there were reasons assigned for the act which made it even more atrocious if possible (revenge for an offence or injury done the negro by the child's father), which should render the punishment inflicted less abhorrent to a just mind. Or, in another case,—the "safety of the community" was the warrant for sending an evidently insane man to the penitentiary. The case was that of George Fowler, who, having been adjudged insane on a trial for attempt at train-wrecking, was confined in the asylum at Austin. He was an *eroto-maniac*, but during his quiet times he was once or twice furloughed and allowed freedom. He would show symptoms of insanity and be returned to the asylum. The people of his county were afraid of him, and deprecated his being turned loose. The last time he was furloughed he had a return or an attack of erotic mania, and he ravished the first woman he saw; an old, feeble white woman, a grand-mother. He attacked her on the public highway, in sight and sound of habitations and of persons passing along the road, dragging her from her buggy. He was tried for rape, and his insanity(which was well established and unquestioned) was again shown to the satisfaction of a jury. Nevertheless he was sentenced to, and is now serving a term in the penitentiary.

A third case (and many could be cited, occurring here in Texas, *since the burning* of the rapist and murderer, Henry Smith, at Paris, Texas), was that of Ed. Nichols, a strong, well developed negro man of twenty. Seeing a young white girl, a Bohemian, of ten or twelve years of age, pass along the road near where he was picking cotton, he attacked and ravished her, using his thumbs (according to the testimony of the girl, who recovered, and at the trial identified him, and the physician, Dr. J. T. Barnwell) for the purpose of enlarging the vulva and vagina, tearing the perineum into the rectum. Dr. Barnwell, who repaired the torn parts, informed me that the negro's organ must have entered the abdominal cavity of the child. The negro had neither friends nor money, but the court assigned counsel to defend him. The question of insanity was not raised, but Mr. Walton, of counsel, informed me that the negro was an imbecile, if not an idiot; that he seemed to not comprehend that he had done anything wrong, showed no emotion when told that unless he would make some excuse upon which his lawyers could try to save him his neck would be broken; remained throughout the trial with a face of blank expressionless stupidity, and when the sentence of death was passed upon him, and he saw that he was an object of interest, a broad grin spread over his face.

Now, in these cases where shall the line be drawn? If George Fowler was insane, were not the other two insane also? Who, in the present state of our knowledge of sexual perversion and its relation to insanity, shall pronounce,—and let go a responsible human brute, a constant menace to a community, or send to the gallows or stake, an *eroto-maniac*, possibly an irresponsible person? or *hang an idiot ?**

The enforcement of the law is supposed to be for the protection of society and the welfare of mankind. It certainly should have for its end something higher, nobler, and more in keeping with an advanced civilization than *revenge*, or, as is alleged with regard to capital punishment, the repression of crime. The aim of jurisprudence should be, in addition to the repression of crime, a removal of the causes that lead to it, and reform, rather than the extermination of the vicious. It should comprehend both therapeusis and prophylaxis in the widest sense; thus, drunkenness should be cured, and intemperance prevented; the drink-demon be banished forever from the homes of men. So with the sexual sins; the offender should be rendered incapable of a repetition of the offense, and the propagation of his kind should be inhibited in the interest of civilization and the well-being of future generations.

---

[*Since this was written this man, Nichols, was convicted and sentenced to death; on appeal the sentence was confirmed, and he is under sentence to be executed December 22, inst.—ED.]

These ends are not fulfilled by hanging, electrocution, or burning at the stake,

It may be answered that capital punishment does fulfill two of the ends; it prevents a repetition of the offense, and stops hereditary transmission; but that it lessens crime cannot be admitted. After the abolition of the death penalty for rape in England the crime greatly increased; but in Texas, although the offense is visited with swift retribution, and often a cruel death is inflicted by a mob; yet rape, and that too, in its most horrible, cruel and revolting form, that of tender young girls, is greatly on the increase. Not a newspaper can be picked up that does not contain the announcement of something of the kind, and it is by no means confined to Texas. Even the horrible execution by fire of the wretch at Paris, Texas, seems to have been forgotten, if it has not, indeed, *acted as a suggestion or excitant*, to that incomprehensible race, the negro, so different from his immediate progenitors of only one or two generations ago.

That capital punishment has failed of its only ostensible object, keeping the criminal instinct in abeyance, has been clearly demonstrated in Texas, and it ought to be abolished. It is to be condemned from every standpoint, and it is gratifying to be assured that in the great State of New York "the subject is being seriously considered," and is, according to Hon. Clark Bell, "nearer accomplishment than ever before." (*Medico-Legal Journal*, June, 1893.) The bungling and revolting electrocutions witnessed there should be ample warrant for its abolishment. Were no other objections to be urged against the death penalty, the fact that innocent persons, and often doubtful ones,—and it may be, *irresponsible* ones,—are sometimes executed, should be fatal to the law and secure its repeal.

In lieu thereof, and as a solution to the most difficult problem in sociology which confronts the learned professions to-day, and as a measure calculated to fulfill all the ends and aims of criminal jurisprudence, castration is proposed. I say "castration" and not "asexualization," because that applies as well to women; and in sexual perversion the woman is usually passive; she cannot commit a rape, at all events (though she can practice sexual abominations that shock morals, wreck health, and worse, can transmit her defects to posterity). In light of the Alice Mitchell case it might be well enough to adopt Dr. Orpheus Evert's suggestion and asexualize all criminals of whatever class.

The suggestion is not new, by any means. Castration has been advocated by numerous writers in various parts of the world, as a punishment for crime; but that it has ever been practiced to any extent anywhere for the purpose of curing mental disorders, or with any intention or thought of arresting the hereditary transmission of either disease or vices of constitution, in short, for the *purpose of prophylaxis as applied to race improvement and the protection of society*, I am not advised. In this country, and recently, several writers have advocated castration. Dr. W. A. Hammond's paper on the subject will be recalled by all present. Dr. Frank Lydston (*Va. Medical Monthly*) in reply to a question from Dr. Hunter McGuire as to the cause of so much rape by negroes in the South, advises castration as a remedy for the evil; and there is much wisdom in the advice. He would castrate the rapist, thus rendering him incapable of a repetition of the offense, and of propagating his kind, and *turn him loose*—on the principle of the singed rat—to be a warning to others. Dr. Lydston says, and very truly, that a hanging or even a burning is soon forgotten; but a negro buck at large amongst the ewes of his flock, minus the elements of manhood, would be a standing terror to those of similar propensities. Dr. Orpheus Everts (*Lancet-Clinic*, March, 1888,) would castrate all convicted criminals, thus arresting the descent of their respective vices of constitution. This is very extreme, and would be advisable, perhaps, were it not that the same objection attaches as to hanging; innocent persons are sometimes convicted of crime, and we might *cut the wrong man*. Dr. Blummer (for Dr. Gardner) *American Journal Insanity*, reports ten cases of castration for epilepsy, all successful; and Dr. White (*loc. cit.*) reports the details of one case of castration, unsuccessfully done for epilepsy thought to be due to reflex action in sexual disturbance, the patient having priapism without desire, and wasting seminal losses.

But as stated, I know of no instance where castration has been systematically practiced for the cure of preverted sexual instinct, or the state of mind resulting from its exercise, or giving origin to it; and certainly no instance where it has been done with any reference to posterity.

In light then of the very evident doubt as to the sanity of those who commit sexual crimes, and therefore, of their responsibility; and particularly as it is impossible in the present state of our knowledge to draw a line and say where, in mental alienation, unsoundness to the extent of irresponsibility for acts exists, I would substitute castration as a penalty for all sexual crimes or misdemeanors, including confirmed masturbation.

Reasoning a *priori*, if a perverted sexual sense be the cause of mental disturbance and unsoundness,—and there can be no doubt that habitual masturbation frequently is,—both cause and effect,—the removal of the glands should restore the equilibrium, on the fundamental principles on which we practice medicine; if it be an expression of diseased mind, as held by Drs. Kellogg, Chaddock and most other authorities, the operation may exert a beneficial influence on the mind; as we know that asexualization often completely changes the character of the individual, and that too, without detriment to the mere physical man. Reasoning by analogy, if the removal of the ovaries will cure hysteria or hystero-epilepsy, as is extensively claimed for properly selected cases, surely we should be warranted in hoping that castration will, by obliterating the sense, relieve some, if not all of the disturbances of the mind.* It would be an advisable hygienic measure in habitual masturbation, whether the practice be cause or effect, by arresting the wasting of vital force by seminal losses, and consequent impairment of physical health.

In light of all these facts, I think we are warranted in at least making the experiment on a scale large enough to test the operation as a therapeutic measure, we know the operation would be prophylactic and protective, both to society and to posterity.

It has occurred to the profession to test Battey's ideas of the curative effect of the removal of the ovaries, for disturbance in the female similar to those in males now under consideration; and the experiment was being made on a large scale in the Pennsylvania hospital by Dr. Joseph Price and others (*Jour. Am. Med. Ass'n* Jan. and Feb., 1893), when such an outcry was raised that it had to be discontinued before any definite results could be formulated. The board of charities, through their "legal member," put a stop to it, on the ground that it is unlawful to unsex a woman without her consent; and being presumably insane, these women could not give their consent. Thus, great

---

* Authors agree that if the testes alone are removed, the power of procreation is destroyed, (aspermatism) but not desire and virility. If the testes and the epididimus, together with part of the cord are removed, desire is abolished and the power of erection is destroyed. (See Ref. H. B. Med. Sci.)

regard was had for the *posterity* of these mentally afflicted creatures.

It does seem that the scheme of our government, and the tendency of our civilization is to foster the criminal and mentally defective classes. A writer on insanity in *Reference Hand-book Medical Sciences*, says (p. 54), "But the openly criminal class, taken as a whole, there can be no doubt has the closest affinities with the insane. * * * Among the savages the strongest survive; but one of the universal attendants of civilization in all large cities is a class of beings degraded physically and mentally, that recklessly propagate a large per cent. of idiots, lunatics and criminals."

The lower animals limit production, and eliminate the weaker by battles between the males for the possession of the female; and certain of the rodents. the squirrel I am told, castrate the young males. But with civilized man the procreative function, and the right to exercise it *ad libitum* seems to be something sacred; it is respected, even in those who have, by their misconduct, outraged society, and forfeited all other rights, civil, religious and political. Is is not a remarkable civilization that will break a criminal's neck, but will respect his testacles?

Rational man will not permit his defective stock to breed; but contrary to reason, common sense, and the best interest of society, will permit the consumptive, the insane, the intemperate, the syphilitic and the criminal to propagate each his kind, under the protection of the law. The insane are a burden and an unsolved problem in every State; a kind of public debt we cheerfully bear, and are going to hand down to posterity bearing compound interest; the cost of maintaining them is a sort of *tribute* we chivalrously pay to the procreative organs and rights of all classes. That our laws need amending there can be no doubt.

Were we called upon for an explanation of this remarkable paradox, we should expect to find it in that peculiar political organization and status that entrusts the making of *all laws* to men who know nothing of the laws of health, nor of the etiology, or the nature of disease, nor of the influence of heredity in race propagation; nothing of sanitation; and are therefore ignorant of the sanitary needs of a people. Those who do know,—or know better than any other class,—physicians,—are rarely ever called in counsel in framing laws for the protection of the public health,—to say nothing of those looking to the bodily and mental status of coming generations. The medical profession has no head. Unlike most other civilized governments, in this country there is no minister of public health, and no authorized source whence our law-makers could derive such information as to enable them to enact wise sanitary laws.

Touching the legality of castrating a patient as a therapeutic measure, I had a conversation recently with Gov. J. S. Hogg of this State. He was for four years attorney-general of Texas, and is distinguished for his great legal ability. He assured me that there is not a doubt of the legal right on the part ot a superintendent of an insane asylum to castrate a patient for mental trouble, if, in his judgment, it be necessary or advisable. He would have the same right to castrate a patient as he would have to bleed him in the arm, or to amputate a limb, or do any other operation.

But it is not alone in asylums that castration should be done. I propose it as a penalty for sexual crimes, to be imposed by the judge, upon the finding of a jury. Let the perpetrator of sexual crimes (or if not classed as crimes,—misdemeanors), presently to be enumerated, *forfeit his right to a posterity!* Castration will be at once in these cases, in all probability, punitive, curative, and preventive; and I hold with Dr. Lydston, that it will have a more powerful restraining effect on the rapist than does hanging, burning at the stake or electrocution.

While we can not hope ever to institute a Sanitary Utopia in our day and generation, it would seem within the legitimate scope and sphere of Preventive Medicine, aided by the enactment and enforcement of suitable laws, to eliminate much that is defective in human genesis, and to improve our race mentally, morally and physically; to bring to bear in the breeding of peoples the principles recognized and utilized by every intelligent stock-raiser in the improvement of his cattle; and in my humble judgment the substitution of castration, as advocated above, for the useless and cruel execution of criminals, is the first step in the reformation. I predict that in twenty years the beneficial results of castration for crimes committed in obedience to a perverted (diseased) sexual impulse will be established and appreciated.

Rape, sodomy, beastiality, pederasty and habitual masturbation should be made crimes or misdemeanors, punishable by forfeiture of all rights, including that of procreation; in short by castration, or castration *plus* other penalties, according to the gravity of the offense.

It is to be hoped that the medical profession will no longer neglect the investigation of a psychological state fraught with so much significance to criminal jurisprudence. The meaning of every phase of perverted sexuality should be understood, not only by physicians, but by jurists. A more free and unreserved intercourse between the two learned professions should be fostered, when with a better understanding of the subject in its relation to crime they can co-operate to secure a modification of existing unsatisfactory laws, and the enactment of others, consonant with a more advanced state of medico-legal knowledge. This we owe to ourselves, if we would not merit reproach; to posterity, if we would secure to future generationsthe full fruits of sanitation in the practice of the great science of preventive medicine.

